# Double Type Detection of Triiodide and Iodide Ions Using a Manganese(III) Porphyrin as Sensitive Compound

**DOI:** 10.3390/s24175517

**Published:** 2024-08-26

**Authors:** Diana Anghel, Camelia Epuran, Ionela Fringu, Ion Fratilescu, Anca Lascu, Ana-Maria Macsim, Vlad Chiriac, Mihaela Gherban, Dana Vlascici, Eugenia Fagadar-Cosma

**Affiliations:** 1 Institute of Chemistry “Coriolan Dragulescu”, Mihai Viteazu Avenue 24, 300223 Timisoara, Romania; danghel@acad-icht.tm.edu.ro (D.A.); ecamelia@acad-icht.tm.edu.ro (C.E.); mcreanga@acad-icht.tm.edu.ro (I.F.); ionfratilescu@acad-icht.tm.edu.ro (I.F.); alascu@acad-icht.tm.edu.ro (A.L.); 2 Institute of Macromolecular Chemistry “Petru Poni”, Grigore Ghica Vodă Alley, No. 41A, 700487 Iasi, Romania; macsim.ana@icmpp.ro; 3 Faculty of Chemistry, Biology, Geography, West University of Timisoara, 4 Vasile Parvan Ave, 300223 Timisoara, Romania; vlad.chiriac@e-uvt.ro; 4 National Institute for Research and Development in Electrochemistry and Condensed Matter, P. Andronescu Street, No. 1, 300224 Timisoara, Romania; mihaelabirdeanu@gmail.com

**Keywords:** manganese porphyrin, iodine species, UV-Vis, potentiometric detection

## Abstract

A paramagnetic A_3_B-type Mn(III)-porphyrin was synthesized and characterized by physical–chemical methods (UV-Vis, FT-IR, ^1^H-NMR spectroscopy). The obtained compound was tested as a sensitive material for the spectrophotometric and potentiometric detection of iodine species. Using UV-Vis spectroscopy, the triiodide anions could be detected with high precision in the concentration interval of 1.02 × 10^−5^ to 2.3 × 10^−5^ M, with an LOD of 9.44 × 10^−6^ M. The PVC-based electrode using DOP as a plasticizer showed a sensitivity toward iodide in a wide concentration range of 1.0 × 10^−5^ to 1.0 × 10^−1^ M, with an LOD of 8.0 × 10^−6^ M. Both methods are simple, low-cost, and efficient for the detection of iodine species in synthetic samples and pharmaceuticals.

## 1. Introduction

Iodine is an essential element in the everyday diet of numerous living beings, acting as a nutrient with protective roles. Its deficiency leads to a decrease in time of thyroid hormones, impairing normal thyroid function [1]. On the other hand, an excess of iodine in children (>300 μg/L, 2.375 × 10^−6^ M) also has a negative health effect [2,3], being related to diseases such as goiter [4], hypothyroidism [5], hyperthyroidism [6], and autoimmune thyroid diseases [7]. Iodine and its derivatives have multiple widespread applications in fields such as medical care [8], nutrition [9], disinfection [10], organic synthesis [11], and agriculture [12,13].

The polyhalogen complex anion ($I_{3}^{-}$), is formed due to the combination of iodide as a nucleophile and iodine as an electrophile [14]. Polyiodide anions contain [I_2k+n_]^n−^ units, possessing various coordination structures [15], which makes them perfect for attachment to the large cations, such as starch, chitosan, cellulose, xylan, silk, and even wool [16]. 

Equations (1)–(4) depict the formation of triiodide anions in solution, starting from dissolved iodine and iodide anions (Equation (1)) [17]. Equation (2) represents the disproportionation reaction of the iodine molecule in water [18], Equation (3) presents the iodate disproportionation [19] and Equation (4) represents the iodine–iodide equilibrium leading to triiodide formation [17].

(1) $$2I+H_{2}O\;\to \;I_{2}+H_{2}O,$$

(2) $$I_{2}+H_{2}O\to HIO+H^{+}+I^{-},$$

(3) $$3HIO\to \;3H^{+}+{IO}_{3}^{-}+2I^{-},$$

(4) $$I_{2}+I^{-}\;↔I_{3}^{-}$$

In triiodide ions, iodine has the oxidation state of −1, an oxidation state referring to the atom’s electronic configuration [20].

The literature data regarding the detection and quantification of iodine species include the colorimetric detection of iodide in water, using the photo-irradiation of samples and starch as a color amplifier, for concentrations above 3 × 10^−5^ M [21]. The drawback of this method would be the necessity of photo-irradiation of the samples. Another method based on color change that quantifies 0.14 mg/L to 6.8 mg/L (1.1 × 10^−6^ M to 5.35 × 10^−5^ M) of iodide in water uses diffuse reflectance spectrophotometry, which measures the oxidized products obtained by the introduction of Oxone^®^ (potassium monopersulfate salt, commercially available, Sigma Aldrich, Merck, Darmstadt, Germany) in the measured samples [22]. The drawback of this method is the need for special equipment, purposely designed for utilization in spacecrafts. The spectrophotometric method for the detection of 20–350 ng/g (1.57 × 10^−7^ M to 2.75 × 10^−6^ M) iodide in water proposed in [23] acts optimally only in a narrow, very acidic pH domain (1.5–2.5).

The colorimetric on-site monitoring of iodide can also be performed using a paper-based kit based on chitosan–lactate-capped silver nanoparticles. The concentration domain ranges from 4.9 × 10^−7^ M to 3.38 × 10^−6^ M. The advantage of the method consists of using a physiological working pH [24].

Using expensive equipment such as an HPLC system coupled with amperometric detection, the authors of [25] were able to simultaneously determine iodine (0.5–25 μg/L) (3.94 × 10^−9^ M to 1.97 × 10^−7^ M) and iodate (1–50 μg/L) (5.71 × 10^−9^ M to 2.8 × 10^−7^ M) in natural water samples.

The triiodide ion itself was detected spectrophotometrically, based on its own absorption intensity, in the concentration domain 5 × 10^−3^ M to 1 × 10^−4^ M, but the pH of the solution must be in the range of 2.0–6.5 [26].

It is well known that porphyrins and metalloporphyrins show excellent prospects for applications in many fields, including detection, due to the large macrocycle-possessing extended aromaticity [27], which favors amazing opto-electric and aggregation properties. Our research group previously provided a simple UV-Vis detection method for triiodide anions, using as a sensitive material a Pt-metalloporphyrin (Pt(II)-5,10,15,20-tetra(4-methoxy-phenyl)-porphyrin) complexed with gold nanoparticles The hybrid material was able to detect triiodide anions in the concentration range of 1.55 × 10^−9^ to 4.3 × 10^−8^ M [28]. The hybrid material used in this paper is expensive, as it requires gold nanoparticles.

Porphyrins bearing carboxylic groups [29] were used for photodynamic therapy (PDT) [30], as building blocks for supramolecular assembling [31], as fluorescent pH sensors [32], in sensing for volatile organic compounds and metal ions [33], and as dye-sensitized solar cells [34]. 

Besides the UV-Vis detection, as a technique that is a simple and very efficient method for detection, potentiometry remains an important and promising field in analytical determinations due to its simplicity, selectivity, and fast response. The potentiometric electrodes are prepared by incorporating any ion-exchanger agents, being very useful for clinical, chemical, or environmental analysis. A porphyrin base and metalloporphyrins have been used as ionophores in potentiometric sensor devices for the detection of cations and halogens (detection limits between 1 × 10^−7^ and 1 × 10^−8^ M) [35], diclofenac (detection limit of 1.5 × 10^−6^ M) [36], thiocyanate (detection limit of 4.2 × 10^−7^ M) [37], and gibberellic acid [38].

Due to their attractive photoelectronic properties, high metal–ligand stability, and various oxidation states, manganese porphyrins draw interest in multiple studies as magnetic materials [39], perovskite solar cells [40], synergistic sonodynamic therapy and ferroptosis in hypoxic tumors [41], gas sensors [42], detectors of ascorbic acid [43], and superoxide dismutase (SOD) mimics [44].

**Figure 1:**
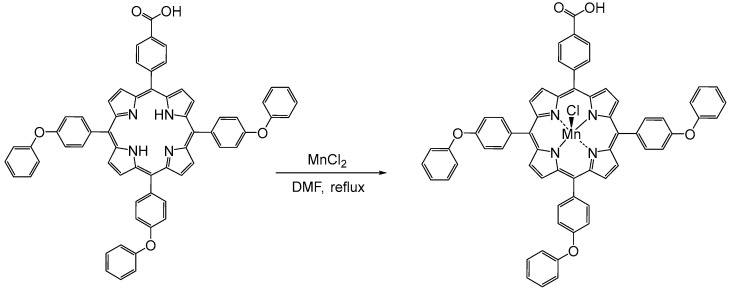
Synthesis of (5-(4-carboxy-phenyl)-10,15,20-tris-(4-phenoxy-phenyl)-porphyrinmanganese(III) chloride (Mn(III)Cl-COOH-TPOPP).

The present work proposes two detection methods, UV-Vis and potentiometric, for triiodide and iodide ions, respectively, from body fluids and pharmaceuticals, using an A_3_B porphyrin metalated with manganese. The Mn(III)-porphyrin was prepared by direct metalation of the porphyrin-free ligand, as represented in Figure 1. The effectiveness of Mn(III)Cl-COOH-TPOPP in both the UV-Vis and potentiometric techniques was demonstrated by developing fast, simple, low-cost, precise and selective methods.

## 2. Materials and Methods

### 2.1. Chemical Reagents 

Tetrahydrofuran (THF), dimethylformamide (DMF), manganese(II) chloride (MnCl_2_), hydrochloric acid (HCl), potassium iodide (KI), bis(2-ethylhexyl)sebacate (DOS), o-nitrophenyloctylether (NPOE), dioctylphtalate (DOP), sodium tetraphenylborate (NaTPB), ethanol, and poly(vinyl)chloride (PVC) of high molecular weight were acquired from Merck (Darmstadt, Germany). Iodine was purchased from Fluka (Basel, Switzerland). Methanol (MeOH) and 4-morpholinoethanesulfonic acid (MES) were bought from Sigma Aldrich (St. Louis, MO, USA). Dichloromethane (CH_2_Cl_2_) was procured from Chimreactiv (Bucharest, Romania).

### 2.2. Manganese Porphyrin (5-(4-Carboxy-phenyl)-10,15,20-tris-(4-phenoxy-phenyl)-porphyrinmanganese(III) Chloride, Mn(III)Cl-COOH-TPOPP, Synthesis

The manganese porphyrin (5-(4-carboxy-phenyl)-10,15,20-tris-(4-phenoxy-phenyl)-porphyrinmanganese(III) chloride, Mn(III)Cl-COOH-TPOPP) was obtained in the laboratory as follows: a solution of 5-(4-carboxy-phenyl)-10,15,20-tris-(4-phenoxy-phenyl)-porphyrin (30 mg, 0.032 mmol) in DMF (100 mL) was treated with a solution of MnCl_2_ (100 mg, 0.806 mmol) in MeOH (10 mL) by dropwise addition at 100 °C. The mixture changed its color from the initial wine-red to brown and then to green (after 5 min) after MnCl_2_ was added. The solution was brought to reflux and stirred for another 4 h. The UV-Vis spectra were recorded every 15 min. The obtained solution (green color) was cooled to room temperature and DMF was removed under a vacuum. The resulting solid was dissolved in CH_2_Cl_2_ with 5 mL MeOH, then washed with 100 mL of HCl (c = 0.05 M) and several times with hot water, dried with Na_2_SO_4_ and concentrated (26 mg obtained, 79.48%) [45]. 

### 2.3. Triiodide Ions Preparation

The solution of triiodide ions was prepared by mixing potassium iodide solved in water (c = 9.5 × 10^−4^ M) and molecular iodine (c = 9.5 × 10^−4^ M). The obtained mixture was heated to 60 °C under vigorous stirring until the iodine was completely solved.

### 2.4. Polymeric Membrane Preparation

The membrane preparation was performed as follows: the amounts of Mn(III)Cl-COOH-TPOPP, PVC, DOS (or DOP, or o-NPOE), NaTPB, and THF (Table 1) were mixed until a blue transparent solution was obtained. The obtained solutions were transferred onto a glass plate of 20 cm^2^, and the THF was allowed to evaporate at room temperature. A piece of membrane (8 mm in diameter and 1 mm thickness) was cut out and assembled on the Fluka electrode body.

All the sensors were soaked in 10^−3^ M potassium iodide solution for 24 h to be conditioned before use.

The measurements were carried out at room temperature using a Hanna Instruments HI8817 pH/mV meter (Cluj, Romania) by setting up the following cell:

Hg|Hg_2_Cl_2_|bridge electrolyte|sample|ion-selective membrane|0.01 M KCl|AgCl, Ag

The potentiometric selectivity coefficients were determined according to the separate solution method [46] by using the experimental EMF values obtained for 10^−3^ M solutions of the tested interfering species. The detection limit of each sensor was established at the point of intersection of the extrapolated linear mid-range and the final low concentration level segments of the calibration plot.

### 2.5. Real Sample Preparation

For the iodide detection from the real sample, two different pharmaceutical tablets were used. Two pills from each solid tablet were dissolved in 50 mL of distilled water. 

### 2.6. Apparatus

For the monitoring of the manganese porphyrin preparation reaction and the detection of triiodide ions, a UV-Vis V-650 JASCO spectrometer (Pfungstadt, Germany) was used, in 1 cm wide quartz cuvettes. The FT-IR spectra were registered on a Jasco 430 instrument in the 400–4000 cm^−1^ range (using KBr pellets) (Hachioji, Tokyo, Japan). All the potentiometric measurements were performed using a Hanna Instruments HI223 pH/mV meter (Cluj, Romania). The ^1^H-NMR spectra were recorded on a Bruker Avance NEO 400 MHz apparatus (Rheinstetten, Germany). For dissolving the samples, deuterated chloroform was used. 

The AFM investigations were recorded on a Nanosurf^®^ EasyScan 2 Advanced Research AFM (Liestal, Switzerland). Measurements were taken by preparing the samples on a silica plate using a stiff (450 μm × 50 μm × 2 μm) piezoelectric ceramic cantilever (spring constant of 0.2 Nm^−1^), with an integral tip oscillated near its resonance frequency of about 13 kHz. All the AFM measurements were performed at room temperature in contact mode. 

## 3. Results and Discussion

In order to better understand the influence of introducing a manganese ion into the porphyrin core, we investigated the obtained compound by various methods, including UV-Vis and FT-IR. Mn(III) porphyrins and their complexes belong to the third type of spectra, the d-hyper type. 

### 3.1. UV-Vis Monitorization of Mn(III)Cl-COOH-TPOPP Synthesis

**Figure 2:**
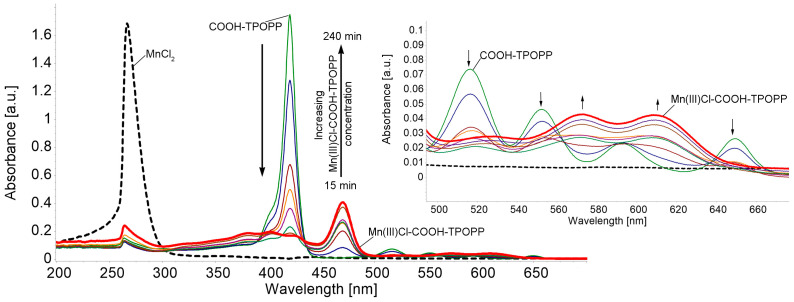
UV-Vis monitoring of Mn(III)Cl-COOH-TPOPP synthesis by direct metalation of porphyrin-base. Detail represents the Q band region of the spectra, along with the changes in the shape, number and position of peaks during metalation.

The UV-Vis monitorization of Mn(III)Cl-COOH-TPOPP synthesis is detailed in Figure 2. 

After the complete metalation of COOH-TPOPP, the UV-Vis spectrum of the resulting manganese porphyrin displays two Q bands that appear between 550 and 650 nm and two Soret bands in the range of 350–440 nm. The intense Soret band shows up red-shifted to 474 nm, unlike the Soret band of the porphyrin base around 420 nm. Due to the presence of additional intense absorption bands extra to the normal-type spectra, the UV-Vis spectra of the synthesized Mn correspond to the hyper type [47].

**Figure 3:**
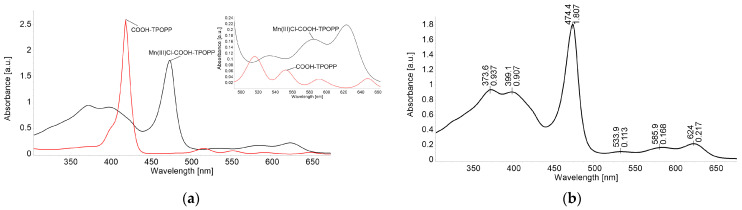
(**a**) Comparative spectra of COOH-TPOPP (c = 3.21 × 10^−6^ M) in THF and Mn(III)Cl-COOH-TPOPP (c = 2.2 × 10^−5^ M) in THF. Q bands’ detail. (**b**) The UV-Vis spectrum of Mn(III)-5-(4-carboxy-phenyl)-10,15,20-tris-(4-phenoxy-phenyl)-porphyrin (Mn(III)Cl-COOH-TPOPP) in THF (c = 2.427 × 10^−5^ M) (λ_max_ [nm] (log ε)): 374 (4.59); 399 (4.57); 474 (4.87); 586 (3.84); and 624 (3.95).

Figure 3a presents the drastic changes in the electronic spectrum that occur during the metalation reaction. It can be seen that the Soret band located at 420 nm of the porphyrin base is decreasing in intensity and is shifted with 54.4 nm as the metalation reaction evolves. The new Soret band located at 474.4 nm, attributed to the manganese porphyrin, is due to the charge transfer from the a_1u_ and a_2u_ orbitals of the porphyrin base to the eg(dπ) orbital of manganese. This band is called the charge transfer band [48].

As the literature describes (presented in Figure 3b) [49], the calculated energy levels for the molecular orbitals of Mn(III)-porphyrins are as follows: a′_2u_(π) < b_2u_(π) < a_1u_(π) < a_2u_(π) < b_2g_(d_xy_) < eg(d_xz_, d_yz_) < a_1g_(d_z2_) < e_g_*(π) < b_1g_(d_x_^2^-_y_^2^). The unique spectral features of Mn(III)-porphyrins may be attributed to π interactions, involving the metal (dπ) and porphyrin and chloride donor orbitals, respectively [50].

### 3.2. Comparison of the FT-IR Spectra of COOH-TPOPP and Mn(III)Cl-COOH-TPOPP

**Figure 4:**
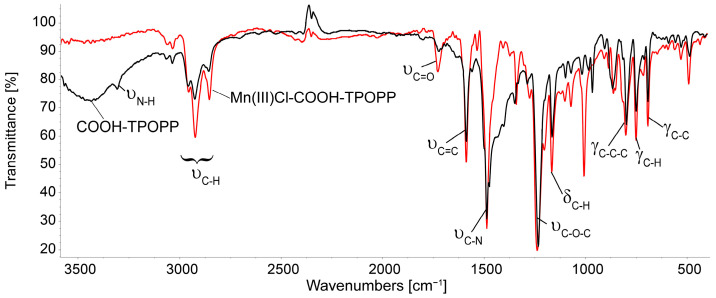
Overlapped FT-IR spectra of COOH-TPOPP and Mn(III)Cl-COOH-TPOPP in KBr pellets.

The IR spectrum of Mn(III)Cl-COOH-TPOPP (Figure 4) shows the characteristic bands of the porphyrin as follows: the peak located at 1587 cm^−1^ corresponds to the stretching vibration of the (C=CPh) bond, the one around 1238 cm^−1^ represents the vibration of the aromatic C–O-C bond, and the porphyrin-specific (C–H_Pyrrole_) bond is vibrating at 751.1 cm^−1^. The band attributed to the N-H stretching, which appears at 3307 cm^−1^ in the COOH-TPOPP, is no longer visible in the spectrum of manganese porphyrin, a fact that demonstrates the complete metalation of the porphyrin base. The band around 1487 cm^−1^ can be related to the C=N stretching vibration in Mn(III)Cl-COOH-TPOPP. The band located at 1007 cm^−1^ is attributed to asymmetric vibrations of the pyrrole ring because δ(C-H) vibrations strongly depend on the nature of the metal [51,52,53]. The bands present at around 2850 cm^−1^ and 2900 cm^−1^ are attributed in both compounds to the symmetrical and asymmetrical aromatic vibrations of C-H bonds [54].

### 3.3. ^1^H-NMR of Mn(III)Cl-COOH-TPOPP Structure

Because the trivalent manganese ion is paramagnetic [55], Mn^3+^ porphyrins have a d^4^ electron configuration with two or more unpaired electrons; therefore, the inner sphere of the porphyrin centered on the metal ion is a blind zone, so some proton ^1^H-NMR signals are too broad to be detected (Figure S1) [56,57].

### 3.4. Optical Detection of Iodide Anions

**Figure 5:**
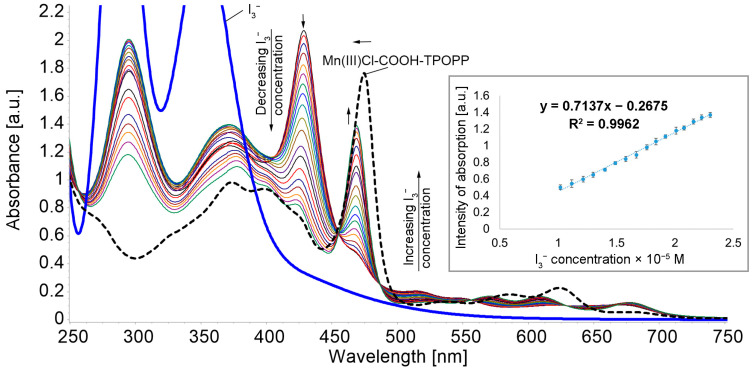
Superposed UV-Vis spectra after adding $I_{3}^{-}$ in H_2_O (9.5 × 10^−4^ M) to Mn(III)Cl-COOH-TPOPP solution in THF (c = 2.5 × 10^−5^ M) (isosbestic point at 454 nm). Linear dependence between the intensity of absorption and the $I_{3}^{-}$ concentration read at 469 nm.

To 3 mL Mn(III)Cl-COOH-TPOPP solution in THF (c = 2.5 × 10^−5^ M), portions of 0.04 mL $I_{3}^{-}$ solution in H_2_O (c = 9.5 × 10^−4^ M) were successively added. After each addition, the mixture was stirred for 60 s and the UV-Vis spectrum was recorded (Figure 5). 

The addition of triiodide ions to the Mn(III)Cl-COOH-TPOPP leads to a hypsochromic shift of the Soret band from 474 nm to 469 nm, with the appearance of a new band around 428 nm. This process occurs due to the interaction between the cationic core of the porphyrins and the negatively charged triiodide ions. The intensity of the new band is decreasing with the increase in the triiodide concentration in the system and the band around 469 nm is increasing in intensity. The linear dependence between the intensity of the absorption read at 469 nm and the triiodide concentration in the interval 1.02 × 10^−5^ to 2.3 × 10^−5^ M has an excellent correlation coefficient of 99.62%. The limit of detection (LOD) was determined as 9.44 × 10^−6^ M (detail in Figure 5). The isosbestic point from 454 nm indicates the formation of intermediate species that are found at equilibrium [58].

#### 3.4.1. Proposed Mechanism for Triiodide Recognition

**Figure 6:**
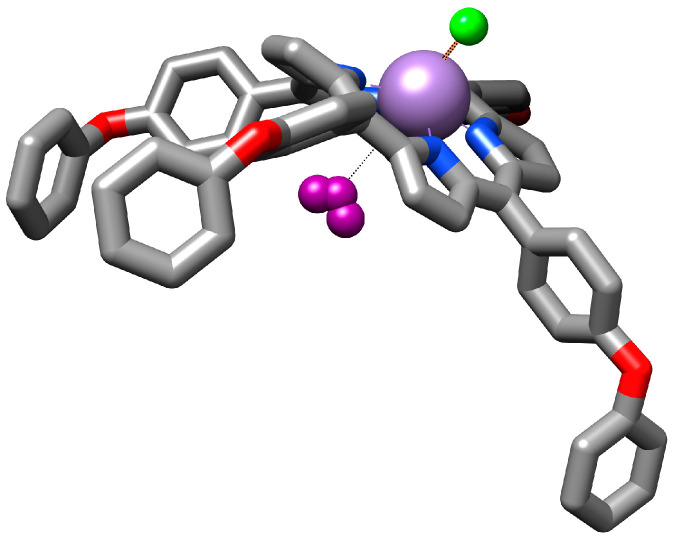
Illustration of the proposed mechanism for triiodide recognition.

The porphyrin base molecule is large and, due to both the presence of the central manganese metal ion, which exerts steric effects upon the macrocycle, and its further interaction with the triiodide anion, it is likely that the whole molecule will adopt an optimized dome-like geometry [59]. The manganese ion is susceptible to increasing its oxidation state to higher values after interaction with the triiodide anions. So, the voluminous triiodide anion can be accommodated as a ligand inside the dome-shaped metalloporphyrin (Figure 6).

#### 3.4.2. Study of the Influence of Potential Interfering Species on the Spectroscopic Detection of Triiodide

To the 3 mL Mn(III)Cl-COOH-TPOPP solution in THF with $I_{3}^{-}$ solution in water (c = 2.29 × 10^−5^ M), 0.1 mL solutions of anions and cations were added at a concentration of 1 × 10^−2^ M. The potential interfering species, such as magnesium perchlorate (MgClO_4_), sodium azotate (NaNO_3_), sodium azotite (NaNO_2_), calcium gluconate (CaGlu), potassium iodide (KI), potassium chloride (KCl) and sodium citrate (Na_3_C_6_H_5_O_7_), were chosen due to their presence in body fluids and pharmaceuticals. After every addition, each sample was stirred for 60 s and then the UV-Vis spectrum was recorded. The potential interfering species have a concentration 100 times higher than the studied analyte.

**Figure 7:**
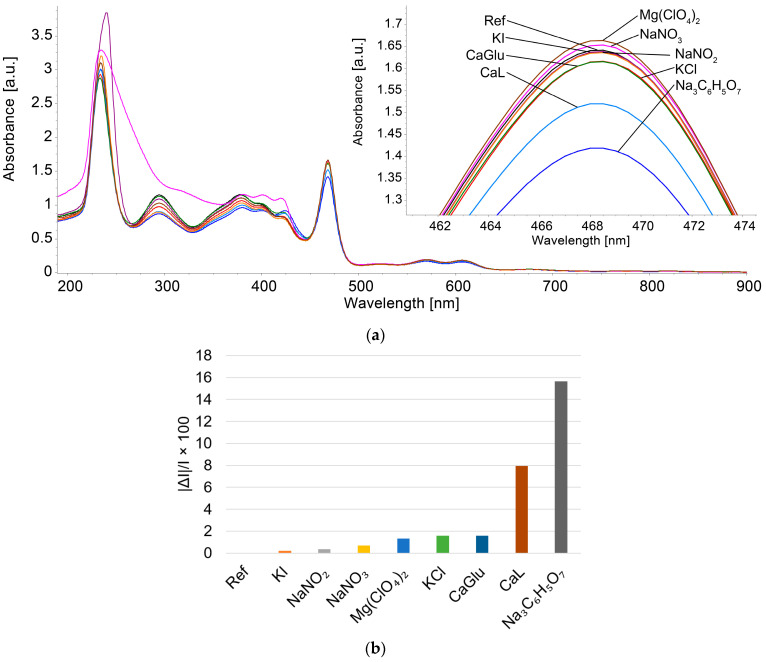
(**a**) Overlapping UV-Vis spectra recorded after adding interfering compounds in the optical detection of $I_{3}^{-}$ with Mn(III)Cl-COOH-TPOPP. (**b**) Graphical representation of the average percentage error in concentrations exceeding 100 times the triiodide ion concentration.

The deviations induced in the intensity of absorption were calculated using the average percentage errors equation (Equation (5)), where I represents the absorption intensity of the sample containing only $I_{3}^{-}$ and ΔI is the difference between I and the absorption intensity of the samples containing $I_{3}^{-}$ mixed with each studied interfering specie [60], as represented in Figure 7b.

|ΔI/I| × 100 (5)

It can be observed that the interfering species studied have no influence on the quantification of triiodide, except for calcium lactate and sodium citrate, which introduce average percentage errors above 8% in the case of concentrations 100 times higher than the concentration of triiodide anion in the analyzed sample. 

### 3.5. AFM Investigation of Mn(III)Cl-COOH-TPOPP before and after Treatment with Triiodide Anion

**Figure 8:**
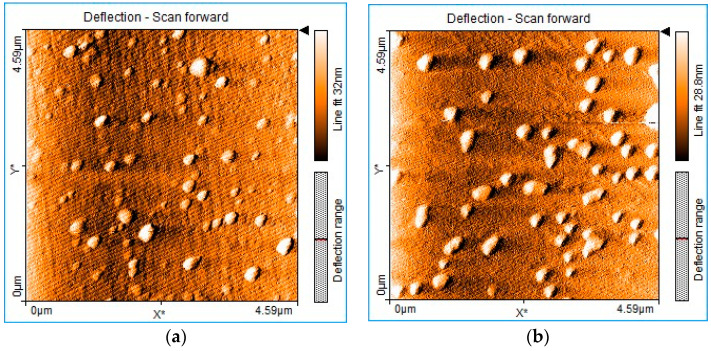
AFM images of (**a**) Mn(III)Cl-COOH-TPOPP and (**b**) Mn(III)Cl-COOH-TPOPP treated with $I_{3}^{-}$.

Atomic force microscopy (AFM) investigations show the changes in the surface morphology after treating Mn(III)Cl-COOH-TPOPP with triiodide anions. The manganese porphyrin deposited from THF (Figure 8a) shows a relatively uneven distribution of haystack-type particles (Figure S2a) with average dimensions of 143–229 nm and average heights of 12.9–17.2 nm (Figure S2b). After manganese porphyrin is treated with triiodide anions (Figure 8b), the architecture of the obtained material changes, showing pyramidal particles (Figure S2c) that are less evenly distributed on the surface, with smaller dimensions around 86–140 nm and a narrow distribution of heights around 8–13 nm (Figure S2d). The decrease in the particle size of Mn(III)Cl-COOH-TPOPP after the exposure to triiodide anions can be explained by the change in conformation of the porphyrin derivative from the saddle type to the dome type, thus gaining a more compact architecture. The interaction followed by the accommodation of the triiodide pyramidal structure inside the dome cavity, as can be seen in Figure 6, after exposure to triiodide anion, favors a compact packaging on the surface.

The surface roughness of the Mn(III)Cl-COOH-TPOPP exposed to triiodide anions measured by AFM slightly decreases to 3.4054 nm as compared to the surface roughness of the solely Mn(III)Cl-COOH-TPOPP, that is, 3.6203 nm (due to its large saddle conformation).

### 3.6. Potentiometric Detection of Iodide Anions

The sensitivity, linearity, and selectivity obtained for a given ionophore depend considerably on the membrane composition, the amount of ionophore, and the nature of the plasticizer and additive. 

In this work, we compared membranes formulated based on three plasticizers [61], o-nitrophenyloctylether (o-NPOE), dioctyl phthalate (DOP) and dioctyl sebacate (DOS), which were selected based on their different dielectric constants as follows: ε = 24, 7 and 4, respectively [62].

**Figure 9:**
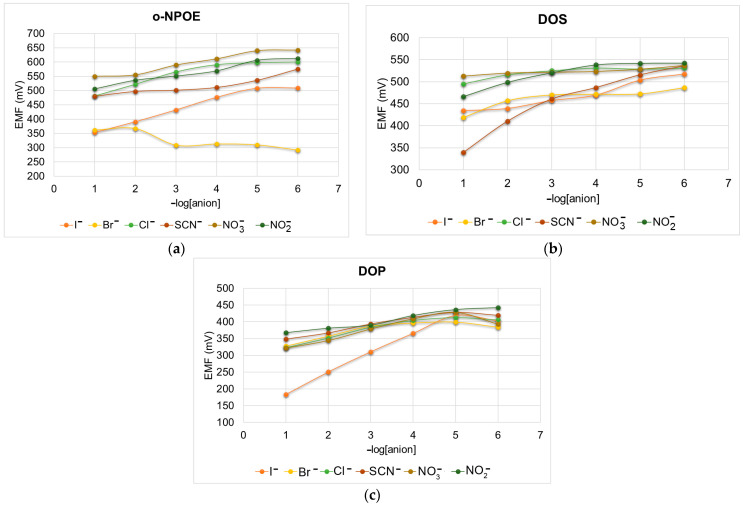
Potentiometric response to different anions using: (**a**) o-NPOE, (**b**) DOS and (**c**) DOP membranes.

The sensors were tested in solutions from 10^−1^ to 10^−6^ M of the following anions: I^−^, Br^−^, Cl^−^, ${NO}_{2}^{-}$, ${NO}_{3}^{-}$, and SCN^−^. The obtained results for each sensor are presented in Figure 9.

As can be seen from Figure 9c, the DOP-plasticized membrane shows an excellent potentiometric response to iodide. Dioctyl phthalate (DOP) or bis(2-ethylhexyl) phthalate is a widely used lipophilic compound, which after being incorporated into the PVC membranes of ion-selective electrodes improves the detection limit and selectivity of the electrodes without decreasing the resistance of the sensing membrane.

**Figure 10:**
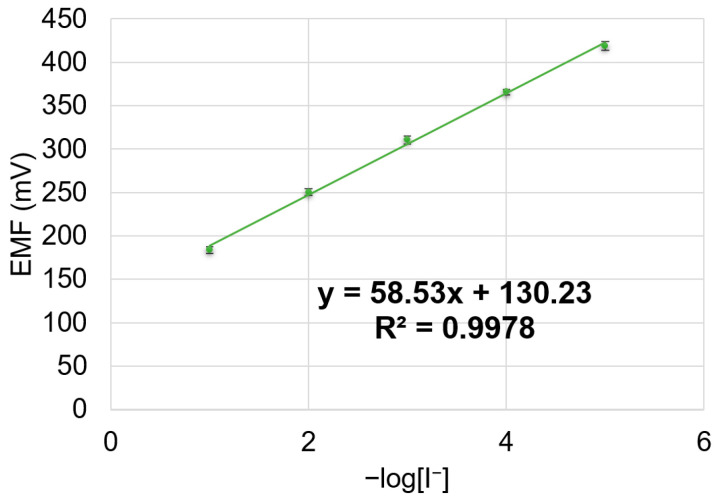
Potentiometric response of DOP-based sensor toward iodide.

The potentiometric answer to iodide of the DOP-plasticized sensor is presented in Figure 10. The experiments were performed in triplicate and the error bars are presented.

As can be seen from Figure 10, the sensor works in a linear range of 10^−1^ to 10^−5^ M, with a near-Nernstian slope of 58.5 mV/decade. The detection limit of the sensor was found to be 8 × 10^−6^ M. 

The response time of the sensor measured from the solution of 10^−3^ M iodide to the solution of 10^−2^ M was about 60 s.

#### 3.6.1. Proposed Mechanism for the Recognition of Iodide Anions by the Mn(III)Cl-COOH-TPOPP Ionophore

The ionophore is immobilized in the plastic PVC membrane, so the analyte representing an anion can lead to a change in the oxidation state of the manganese ion from +3 to +4 or even to +5, so the anion can be bonded to the metal ion in the porphyrin molecule.

#### 3.6.2. Study of Selectivity Coefficients

The selectivity coefficients are a very important part of potentiometric studies, confirming the applicability for the determination of species.

The selectivity coefficients of iodide are presented in Table 2 and are calculated at a 10^−3^ M concentration, with Equation (6) [63], showing no interference and good values. The potential response of the I^−^ ion-selective electrode toward bromide, chloride, nitrite, nitrate and thiocyanate was determined by using the separate solution method.

(6) $$logK_{A,B}^{pot}=\frac{\left(E_{B}-E_{A}\right)\times z_{A}\times F}{R\times T\times ln10}+\left(1-\frac{z_{A}}{z_{B}}\right)\times lga_{A}$$

where *E_A_* and *E_B_* are the potential values of the primary and interfering anions, *a_A_* is the activity, *z_A_* and *z_B_* are the charge numbers of primary and interfering anions, and *R*, *T* and *F* have their usual meaning.

The selectivity order was found to be thiocyanate > chloride > nitrite > bromide > nitrate. 

The sensor presents very good values for the selectivity coefficients.

#### 3.6.3. Analytical Application 

The potentiometric iodide-selective sensor developed by us was successfully used to determine iodide in real and synthetic samples, as can be seen from Table 3.

#### 3.6.4. Potentiometric Determination of Iodide Ions Using Different Sensing Materials

For the potentiometric detection of iodide, various ionophores were used, as presented in Table 4. In the case of using N,N′-disalicylidene-1,2-phenylendiamine dianion as an ionophore, the potentiometric method is suitable to measure the iodide concentration from seawater reservoirs [64]. A tripodal ionophore compound can be used in the potentiometric quantification of iodide in food and biological samples based on the supramolecular halogen-bonding interaction [65]. Screen-printed electrodes were also employed in the potentiometric detection of iodide in seawater; for example, an N-(4,6-Dimethyl-pyrimidin-2-yl)-4-[(2-hydroxybenzylidene)amino] benzene sulfonamide copper(II) dihydrate complex [66] was incorporated into carbon paste. The obtaining of this electrode requires tedious work. Another method for the potentiometric detection of iodide is based on flow injection analysis [67], which provides good results, but the device is not readily available. Another method for the detection of iodide based on imidazolidine-2tion as an ionophore, with a wide working pH range (3.0–10.5), can be used for industrial samples [68]. Aslaner et al. used a dichloro[1,1′-bis(diphenylphosphino)ferrocene]palladium(II) complex as an ionophore [69]. The large-scale application of this method could be an impediment because palladium is a rare and very expensive metal. All the presented ionophores show similar results with our manganese porphyrin, thus confirming the viability of the proposed sensitive material.

## 4. Conclusions

A paramagnetic (5-(4-carboxy-phenyl)-10,15,20-tris-(4-phenoxy-phenyl)-porphyrinmanganese(III) chloride (Mn(III)Cl-COOH-TPOPP) was synthesized by a simple and efficient metalation reaction of the porphyrin base and the obtained compound was characterized by different physical–chemical methods (UV-Vis, FT-IR, ^1^H-NMR spectroscopy). The obtained compound was tested as a sensitive material for spectrophotometric and potentiometric detection of iodine species. Using UV-Vis spectroscopy, the triiodide anions could be detected with high precision in the 1.02 × 10^−5^ to 2.3 × 10^−5^ M concentration interval, with an LOD of 9.44 × 10^−6^ M, with an excellent correlation coefficient of 99.62%. The PVC-based electrode using DOP as a plasticizer showed a sensitivity toward iodide in a wide concentration range of 1.0 × 10^−5^ to 1.0 × 10^−1^ M, with an LOD of 8.0 × 10^−6^ M and a near-Nernstian slope of 58.5 mV/decade and a response time of 60 s. The mechanisms of recognition are based on the capacity of the manganese ion to change its oxidation state in the presence of the monovalent anionic species. Both methods are simple, low-cost, and efficient for the detection of iodine species in synthetic samples and pharmaceuticals.

## Figures and Tables

**Table 1:** Quantities of components used for the preparation of the membranes.

Ionophore (g)	Plasticizer (g)			NaTPB (g)	PVC	THF (mL)
0.0045	DOP	o-NPOE	DOS	0.0009	0.1485	2
	0.303					
		0.288				
			0.333			

**Table 2:** Selectivity coefficients for iodide ions’ potentiometric detection.

Sensor	Anion	Selectivity Coefficients (10^−3^ M)
DOP	$I^{-}$	0.00
	${Br}^{-}$	−3.12
	${Cl}^{-}$	−2.84
	${NO}_{3}^{-}$	−3.26
	${NO}_{2}^{-}$	−3.07
	${SCN}^{-}$	−2.78

**Table 3:** Determination of iodide from real samples.

Samples	Sample Amount Labeled (mg)	Potentiometric Detection ^a^ (mg)
Iod Forte 600 (Pharmapharm, Targu-Mures, Romania)	0.600	0.597 ± 0.2
KI + Silymarin (Arena Group SA, Bucuresti, Romania)	1	0.95 ± 0.1
Synthetic sample	100	98 ± 0.9

^a^ Average of determinations in three samples.

**Table 4:** Ionophores/electrodes used in the potentiometric determinations of iodide ions.

Ionophore/Electrode	Detection Limit (mol/L)	Detection Domain (mol/L)	Ref.
N,N′-disalicylidene-1,2-phenylendiamine dianion	1.0 × 10^−5^	10^−5^–1	[64]
5,5′-(((5-((1-benzyl-5-iodo-1H-1,2,3-triazol-4-yl)methoxy)-1,3-phenylene)bis(oxy))bis(methylene))bis(1-benzyl-4-iodo-1H-1,2,3-triazole)	1.25 × 10^−6^	10^−9^–10^−1^	[65]
N-(4,6-Dimethyl-pyrimidin-2-yl)-4-[(2-hydroxybenzylidene)amino] benzene sulfonamide copper(II) dihydrated complex	3.2 × 10^−6^	-	[66]
Combined iodide commercial electrode (Hanna, RI, USA)	1.39 × 10^−6^	2.5 × 10^−6^–1.0 × 10^−3^	[67]
Imidazolidine-2tion	7 × 10^−7^	1 × 10^−6^–1 × 10^−1^	[68]
Dichloro[1,1′-bis(diphenylphosphino)ferrocene]palladium(II) complex	2.9 × 10^−8^	1.0 × 10^−6^–1.0 × 10^−1^	[69]
Mn(III)Cl-COOH-TPOPP namely 5-(4-carboxy-phenyl)-10,15,20-tris-(4-phenoxy-phenyl)-porphyrinmanganese(III) chloride	8 × 10^−6^	1.0 × 10^−5^–1.0 × 10^−1^	This work

## Data Availability

The data presented in this study are available on request from the first or the corresponding author.

## Supplementary Materials

The following supporting information can be downloaded at https://www.mdpi.com/article/10.3390/s24175517/s1, Figure S1: ^1^H-NMR spectrum of (5-(4-carboxy-phenyl)-10,15,20-tris-(4-phenoxy-phenyl)-porphyrinmanganese(III) chloride in deuterated chloroform; Figure S2: Three-dimensional AFM images and height distribution of (a,b) Mn(III)Cl-COOH-TPOPP and (c,d) Mn(III)Cl-COOH-TPOPP treated with triiodide anions.
