# Supplementary material for: Double Type Detection of Triiodide and Iodide Ions Using a Manganese(III) Porphyrin as Sensitive Compound
Source: Sensors (Basel). 2024 Aug 26;24(17):5517. doi: 10.3390/s24175517 (PMC11397875; doi:10.3390/s24175517)
Supplement: Supplementary file 1 [file sensors-24-05517-s001.zip › sensors-3157799-supplementary.pdf]

Supplementary material

Double type detection of triiodide and iodide ions using a manganese(III) porphyrin as a sensitive compound

Diana Anghel [1], Camelia Epuran [1], Ionela Fringu [1], Ion Fratilescu [1], Anca Lascu [1], Ana-Maria Macsim [4], Vlad Chiriac [2], Mihaela Gherban [3], Dana Vlascici [2,*] and Eugenia Fagadar-Cosma [1,*]

[1] Institute of Chemistry "Coriolan Dragulescu", Mihai Viteazu Avenue 24, 300223 Timisoara, Romania; danghel@acad-icht.tm.edu.ro (D.A.); ecamelia@acad-icht.tm.edu.ro (C.E.); mcreanga@acad-icht.tm.edu.ro (I.F.); ionfratilescu@acad-icht.tm.edu.ro (I.F.); alascu@acad-icht.tm.edu.ro (A.L.)

[2] Faculty of Chemistry, Biology, Geography, West University of Timisoara, 4 Vasile Parvan Ave, 300223 Timisoara, Romania; dana.vlascici@e-uvt.ro (D.V.); vlad.chiriac@e-uvt.ro (V.C.)

[3] National Institute for Research and Development in Electrochemistry and Condensed Matter, P. Andronescu Street, No. 1, 300224 Timisoara, Romania; mihaelabirdeanu@gmail.com (M.B.)

[4] Institute of Macromolecular Chemistry "Petru Poni", Grigore Ghica Vodă Alley, No. 41A, 700487 Iasi, Romania; macsim.ana@icmpp.ro

* Correspondence: efagadar@yahoo.com or efagadarcosma@acad-icht.tm.edu.ro (E. F.-C.), dana.vlascici@e-uvt.ro (D.V.)

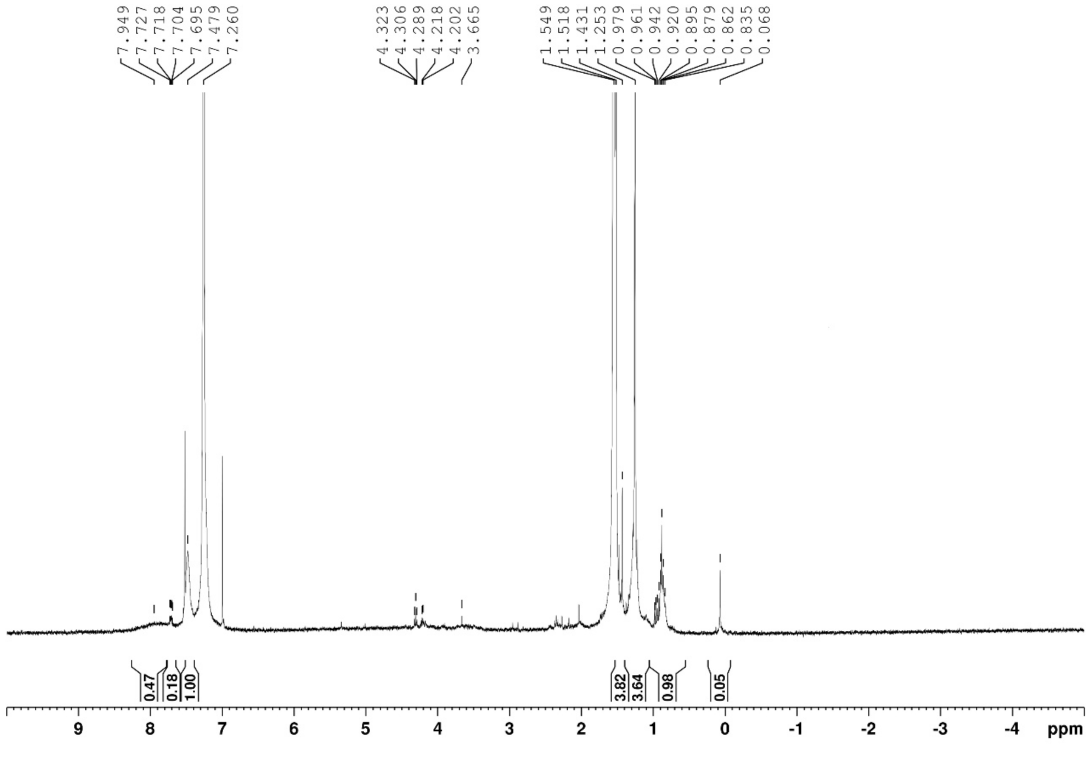

Figure S1. ^{1}H-NMR spectrum of (5-(4-carboxy-phenyl)-10,15,20-tris-(4-phenoxy-phenyl)-porphyrinmanganese(III) chloride in deuterated chloroform.

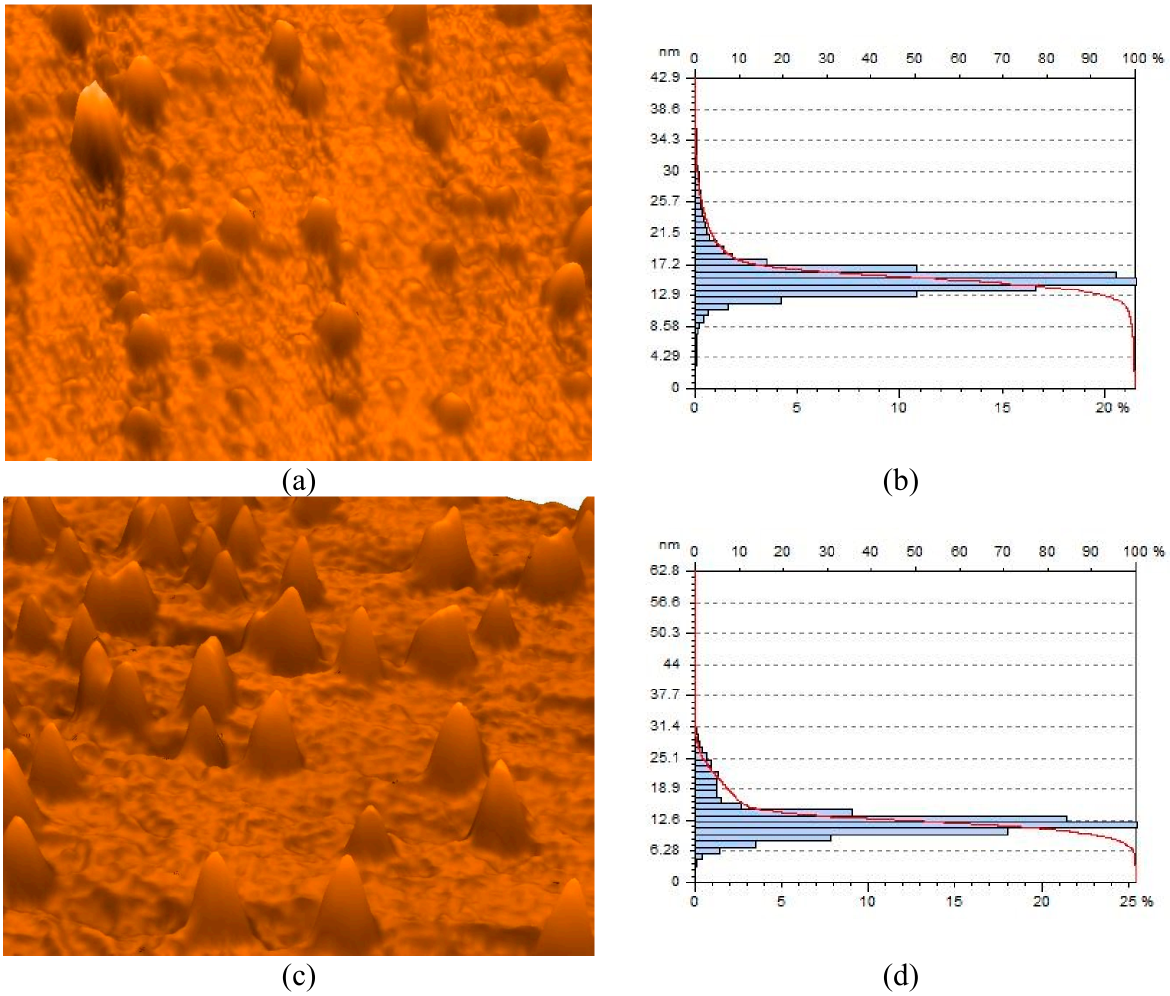

Figure S2. Three-dimensional AFM images and height distribution of (**a,b**) Mn(III)Cl-COOH-TPOPP and (**c,d**) Mn(III)Cl-COOH-TPOPP treated with triiodide anions.
